# Ursodeoxycholic acid versus placebo in the treatment of women with intrahepatic cholestasis of pregnancy (ICP) to improve perinatal outcomes: protocol for a randomised controlled trial (PITCHES)

**DOI:** 10.1186/s13063-018-3018-4

**Published:** 2018-11-27

**Authors:** Lucy C. Chappell, Jenny Chambers, Peter H. Dixon, Jon Dorling, Rachael Hunter, Jennifer L. Bell, Ursula Bowler, Pollyanna Hardy, Edmund Juszczak, Louise Linsell, Catherine Rounding, Anne Smith, Catherine Williamson, Jim G. Thornton

**Affiliations:** 1 King’s College London, London, UK; 2 ICP Support, Sutton Coldfield, UK; 3 Dalhousie University, Halifax, Canada; 4 University College London, London, UK; 5 National Perinatal Epidemiology Unit Clinical Trials Unit, University of Oxford, Oxford, UK; 6 Birmingham Clinical Trials Unit, University of Birmingham, Birmingham, UK; 7 University of Nottingham, Nottingham, UK

**Keywords:** Cholestasis, Pregnancy, Ursodeoxycholic acid, Perinatal, Stillbirth

## Abstract

**Background:**

Intrahepatic cholestasis of pregnancy (ICP) is the most common liver disorder specific to pregnancy and presents with maternal pruritus, raised concentrations of serum bile acids and abnormal liver function tests. ICP is associated with increased rates of spontaneous and iatrogenic preterm labour, fetal hypoxia, meconium-stained amniotic fluid and intrauterine death. Some clinicians treat ICP with ursodeoxycholic acid (UDCA) to improve maternal pruritus and biochemical abnormalities. However, there are currently no data to support the use of UDCA to improve pregnancy outcome as none of the trials performed to date have been powered to address this question.

**Methods:**

The PITCHES trial is a triple-masked, placebo-controlled randomised trial, to evaluate UDCA versus placebo in women with ICP between 20 + 0 to 40 + 6 weeks’ gestation. The primary objective of the trial is to determine if UDCA treatment of women with ICP between 20 + 0 and 40 + 6 weeks’ gestation reduces the primary perinatal outcome: a composite of perinatal death (as defined by in utero fetal death after randomisation or known neonatal death up to 7 days) or preterm delivery (less than 37 weeks’ gestation) or neonatal unit admission for at least 4 h (from infant delivery until hospital discharge). The secondary objectives of the trial are (1) to investigate the effect of UDCA on other short-term outcomes for both mother and infant and (2) to assess the impact of UDCA on health care resource use, in terms of the total number of nights for mother and infant, together with level of care.

**Discussion:**

Current practice in the UK at the time of trial commencement for the treatment of ICP is inconsistent, with some units routinely prescribing UDCA, others prescribing very little and the remainder offering it variably. Our previous pilot trial of UDCA in women with ICP demonstrated that the trial would be feasible, and the research question remains active and unanswered. Results are highly likely to influence clinical practice, through direct management and impact on national and international guidelines.

**Trial registration:**

ISRCTN registry, ID: ISRCTN91918806. Prospectively registered on 27 August 2015.

**Electronic supplementary material:**

The online version of this article (10.1186/s13063-018-3018-4) contains supplementary material, which is available to authorized users.

## Background

Intrahepatic cholestasis of pregnancy (ICP), also called obstetric cholestasis, is the most common liver disorder specific to pregnancy. It presents with maternal pruritus, raised concentrations of serum bile acids and abnormal liver function tests. The maternal symptoms typically resolve postpartum, but affected women have an increased risk of hepatobiliary disease in later life [1]. ICP is associated with increased rates of spontaneous and iatrogenic preterm labour, fetal hypoxia and meconium-stained amniotic fluid [2–4]. There are also reports of increased rates of intrauterine death [5–7], although the incidence is low [4, 8]. Some clinicians treat ICP with ursodeoxycholic acid (UDCA) [9, 10] to improve maternal pruritus and biochemical abnormalities. However, there are currently no data to support the use of UDCA to improve pregnancy outcome as none of the trials performed to date have been powered to address this question.

UDCA is a naturally occurring bile acid that is present in small amounts in humans. It is relatively hydrophilic and has several actions that result in improvement of cholestasis. It increases biliary bile-acid excretion by post-translational modification of hepatic bile-acid transporters, enhances renal bile-acid excretion and has anti-apoptotic effects [11]. UDCA improves outcomes in primary biliary cirrhosis in addition to maternal symptoms in ICP [12]. Adverse events are only infrequently reported in pregnancy [13]; some women experience gastrointestinal side-effects (such as diarrhoea), but in the largest trial to date there were no significant differences in the incidence of reported side-effects in UDCA and placebo groups [2]. Other therapeutic options in ICP include rifampicin, cholestyramine, *S*-adenosyl methionine, guar gum and dexamethasone, but the small studies of these drugs in women with ICP have not consistently shown that they improve maternal symptoms or serum bile-acid concentrations [3].

The main clinical research question is whether adverse pregnancy outcomes can be reduced in women with ICP by treatment with UDCA. This is a subject of intense debate. The current UK guideline (from the Royal College of Obstetricians and Gynaecologists) on the management of ICP describes the evidence relating to the use of UDCA and states that ‘UDCA improves pruritus and liver function in women with obstetric cholestasis’ but ‘women should be informed of the lack of robust data concerning protection against stillbirth and safety to the fetus or neonate’. The guideline concludes that: ‘as the pathophysiology of obstetric cholestasis and the mechanism of fetal demise are uncertain, the possible role of UDCA is unclear. Further larger studies are required to determine this’. The latest updated Cochrane review [13] judged many of the trials of UDCA in ICP to be at moderate to high risk of bias. Trials to date have lacked power to demonstrate whether UDCA is fetoprotective, with numbers of participants and adverse events too small to enable the recommendation of UDCA. The Cochrane review concluded that larger trials of UDCA to determine fetal benefits or risks are needed.

If UDCA is found to be beneficial in ameliorating adverse perinatal outcomes, once published these results would be highly likely to lead to an immediate change in clinical practice, through individual choice of clinicians and women, and through changing national/international guidelines.

The PITCHES trial is a triple-masked, placebo-controlled randomised trial to evaluate UDCA versus placebo in women with ICP between 20^+ 0^ to 40^+ 6^ weeks’ gestation. The primary objective of the trial is to determine if UDCA treatment of women with ICP between 20^+ 0^ and 40^+ 6^ weeks’ gestation reduces adverse perinatal outcomes up to infant hospital discharge. The secondary objectives of the trial are (1) to investigate the effect of UDCA on other short-term outcomes for both mother and infant and (2) to assess the impact of UDCA on health care resource use, in terms of the total number of nights for mother and infant, together with level of care.

## Methods

### Study setting

The study will be conducted in approximately 30 consultant-led maternity units in England and Wales. A list of participating study sites is available on the study website: www.npeu.ox.ac.uk/pitches.

### Inclusion criteria

Women will be considered eligible for inclusion into the trial if they fit the following criteria:

- ICP (pruritus with a raised serum bile acid above the upper limit of normal for the local laboratory)
- 20^+ 0^ to 40^+ 6^ weeks’ gestation on day of randomisation (see note below on gestational age)
- No known lethal fetal anomaly
- Singleton or twin pregnancy
- Aged 18 years or over
- Able to give written informed consent

### Exclusion criteria

Women will be excluded from the trial if:

- A decision has already made for delivery within the next 48 h
- There is a known allergy to any component of the UDCA or placebo tablets
- there is a triplet or higher-order multiple pregnancy

### Intervention

The Investigational Medicine Product (IMP) is ursodeoxycholic acid (UDCA) or matching placebo, manufactured and supplied by Dr. Falk Pharma, GmBH. The most recent Summary of Product Characteristics for UDCA can be found at http://www.medicines.org.uk/emc/medicine/27444. Possible side-effects include gastrointestinal disorders (reported as common, i.e. ≥ 1/100 to < 1/10 patients) or skin and subcutaneous disorders (reported as very rare, i.e. < 1/10,000 patients), as listed in the Summary of Product Characteristics.

#### Formulation and packaging

In the treatment arm, each film-coated tablet contains the active ingredient: 500 mg UDCA and the inactive ingredients of magnesium stearate, polysorbate 80, providone K 25, microcrystalline cellulose, colloidal anhydrous silica, crospovidone and talc. In the control arm, a matching placebo tablet, identical in colour and shape to the treatment arm contains the inactive ingredients: magnesium stearate, polysorbate 80, providone K 25, microcrystalline cellulose, colloidal anhydrous silica, crospovidone and talc. The coating ingredients for both are talc, hypromellose and macrogol 6000. The IMP will be packaged into high-density polyethylene bottles, with 32 tablets per bottle, and will be administered orally. The IMP does not require any special storage conditions.

#### Dosing

The starting dose will be 1000 mg daily (500 mg twice a day), increased in increments of 500 mg per day every 3–14 days if there is no biochemical or clinical improvement, based on clinical decision, to a maximum of 2000 mg per day. The dose of IMP may be reduced to 500 mg daily. Divided doses will be spread evenly throughout the day. There is no need to take it with or without food and this will be left to participant preference. The IMP will be continued until delivery. The duration of treatment will range from 1 day to a maximum of 22 weeks, for a participant randomised at 20 weeks’ gestation who does not deliver until 42 weeks. At each antenatal follow-up visit with a member of the research team, women will be asked the percentage of IMP that they have taken since their last appointment and this will be recorded.

### Study procedures

#### Recruitment, eligibility and consent

Members of the research team will provide a full verbal explanation and written description of the trial to women who meet the inclusion criteria (as above). The woman will be given sufficient time to consider the information, and to decide whether she will participate in the trial. Written informed consent will be sought from the woman and taken by an appropriately trained physician. Baseline data, including all demography, serum bile-acid concentrations and liver function tests, together with an itch visual analogue score (worst itch in the previous 24 h) completed by the woman will be entered on a web-based database by members of the research team at the time of study enrolment. On completion of these details, the database will issue a pack number to the local hospital pharmacy for dispensing.

**Fig. 1:**
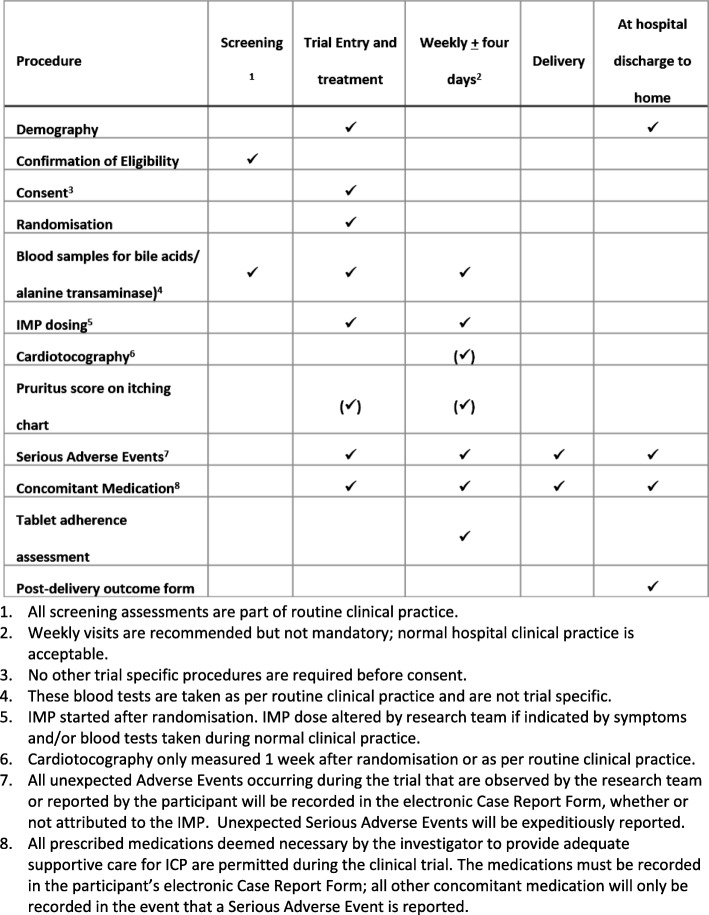
Schedule of participant enrolment, interventions and assessments in the trial. 1. All screening assessments are part of routine clinical practice. 2. Weekly visits are recommended but not mandatory; normal hospital clinical practice is acceptable. 3. No other trial-specific procedures are required before consent. 4. These blood tests are taken as per routine clinical practice and are not trial specific. 5. Investigational Medicinal Product (IMP) started after randomisation. IMP dose altered by the research team if indicated by symptoms and/or blood tests taken during normal clinical practice. 6. Cardiotocography only measured 1 week after randomisation or as per routine clinical practice. 7. All unexpected adverse events occurring during the trial that are observed by the research team or reported by the participant will be recorded in the electronic Case Report Form, whether or not attributed to the IMP. Unexpected serious adverse events will be expeditiously reported. 8. All prescribed medications deemed necessary by the investigator to provide adequate supportive care for ICP are permitted during the clinical trial. The medications must be recorded in the participant’s electronic Case Report Form; all other concomitant medication will only be recorded in the event that a serious adverse event is reported

Participants will be reviewed at routine care clinic visits until delivery. Serum bile acids and liver function tests will be monitored according to usual clinical practice. The woman will be asked for a value of the worst itch she felt during the previous 24 h. The Investigational Medicinal Product (IMP) dose will be altered at the discretion of the responsible clinician. If maximal doses of the IMP have been reached, consideration can be given to the addition of other therapy, e.g. rifampicin, in addition to the trial therapy, without breaking the allocation code. The remainder of antenatal care, in particular the timing and mode of delivery will be left to the discretion of the responsible clinician. A schedule of participant enrolment, interventions and assessments in the trial is shown in Fig. 1.

A woman will be free to withdraw from the clinical trial at any time without the need to provide any reason or explanation; and that this decision will not impact on any aspect of her ongoing clinical care. Women who withdraw will be asked if they are willing for their outcomes to be collected by case note review. If there is sufficient time within the existing study time-line, additional participants will be recruited up to the number of women who discontinued the intervention or withdrew.

Outcomes will be recorded on the web-based database through case note review by trained researchers after discharge of the mother and baby.

The end of the trial will be defined as the date when the trial database is locked. An end-of-trial declaration will be made to the Medicines and Healthcare products Regulatory Agency and the approving Research Ethics Committee.

### Outcomes

The primary perinatal outcome is a composite of perinatal death (as defined by in utero fetal death after randomisation or known neonatal death up to 7 days) or preterm delivery (less than 37 weeks’ gestation) or neonatal unit admission for at least 4 h (from infant delivery until hospital discharge). Each infant will only be counted once within this composite.

The secondary maternal outcomes are:

- Maternal serum concentration (between randomisation and delivery) of the following biochemical indices of disease: bile acids; alanine transaminase; aspartate transaminase; total bilirubin; gamma glutamyl transferase
- Itch between randomisation and delivery, measured by the worst episode of itch over the past 24 h (millimetres on a visual analogue scale, assessed at clinic visits)
- Gestational diabetes mellitus
- Mode of onset of labour
- Estimated blood loss after delivery

The secondary short-term perinatal outcomes are:

- In utero fetal death after randomisation
- Preterm delivery (less than 37 weeks’ gestation)
- Known neonatal death up to 7 days
- Neonatal unit admission for at least 4 h until infant hospital discharge
- Mode of delivery classified as spontaneous vaginal, instrumental vaginal or caesarean
- Total number of nights in the neonatal unit
- Birth weight
- Birth- weight centile
- Gestational age at delivery
- Presence of meconium
- APGAR score at 5 min
- Umbilical arterial pH at birth

The following secondary outcomes will be described only and no formal statistical analysis comparing groups will be conducted:

#### Maternal

- Maximum dose of trial medication required
- Need for additional therapy for cholestasis
- Assessment of myometrial contractions by cardiotocography approximately 1 week (3–14 days) post randomisation
- Reason for induction or pre-labour caesarean section
- Maternal death

#### Perinatal

- Known neonatal death up to 28 days
- Number of nights in each category of care (intensive, high-dependency, special, transitional and normal)
- Need for supplementary oxygen prior to discharge
- Number of days when supplemental oxygen is required
- Need for ventilation support
- Abnormal cerebral ultrasound scan
- Confirmed sepsis (positive blood or cerebrospinal fluid cultures)
- Necrotising enterocolitis (Bell’s stages 2 and 3)
- Seizures (confirmed by electroencephalography or requiring anticonvulsant therapy)
- Encephalopathy (treated with hypothermia)
- Other indications and main diagnoses resulting in neonatal unit admission for at least 4 h

The following health resource use post enrolment will be captured for economic analysis:

- Maternal: total number of nights (antenatal, intrapartum and postnatal) together with level of care including adult Intensive Care Unit; mode of delivery
- Infant: total number of nights for the infant in the neonatal unit, together with level of care (e.g. neonatal Intensive Care Unit)
- The cost of UDCA in the intervention group

All primary and secondary outcomes will be considered up to infant discharge home (from the hospital where delivered) or transfer to another hospital.

### Sample size

We will recruit 580 women in total; this will allow for the possibility of 5% of infants being lost to follow-up and is a conservative estimate given that some women will have twin pregnancies. The sample size is informed by the most recent Cochrane meta-analysis [13]. This includes the trials reported in the previous meta-analysis [14] with the addition of the largest trial published in 2012 by our group [2]. From these data, we estimate the event rate for the primary outcome (a composite of perinatal death or preterm delivery (less than 37 weeks’ gestation) or neonatal unit admission) for infants of untreated women as 40% with a plausible and relevant reduction to 27% for infants of women treated with UDCA, corresponding to an absolute risk reduction of 13% and a risk ratio (RR) of 0.675. This is conservative compared with the effect sizes seen in the Cochrane meta-analysis [13] for the three individual endpoints (RR 0.31, 0.46 and 0.48 for perinatal death, preterm delivery and neonatal unit admission, respectively). Five hundred and fifty infants of women with ICP (275 per group) are required to have a 90% chance of detecting, as significant at the two-sided 5% level, a reduction in the primary outcome measure from 40% in the control group to 27% in the treated group. Allowing for 5% lost to follow-up requires a total sample size of 580 infants (290 per group).

This number will also allow us to look at the components of the composite endpoints: a trial assessing 550 infants will have 89% power to demonstrate a reduction in neonatal unit admission rates from 17 to 8%, and 99% power for a reduction in prematurity from 41 to 23% (based on the Cochrane meta-analysis [13]), both effect sizes of the same magnitude as that demonstrated in our previous trial [2]. We do not anticipate enough perinatal deaths to detect reliably any plausible treatment effect, but we have included this due to its clinical importance and will report it separately.

The trial will be undertaken in approximately 30 consultant-led maternity units in England and Wales to achieve this sample size in the anticipated time-frame. Our previous pilot trial [2] has confirmed that women and clinicians are willing to participate in this randomised controlled trial and we have used recruitment estimates from this trial, together with trial management experience and expertise of the Co-investigator Group to inform strategies to meet the required target size.

### Randomisation

The allocation ratio of intervention (UDCA) to control (placebo) arms will be 1:1. Randomisation will be managed via a secure web-based randomisation facility. A minimisation algorithm will be used to ensure balance between the groups with respect to study centre (approximately 30 centres), gestational age at randomisation (< 34, 34 to < 37, ≥ 37 weeks’ gestation), single versus multi-fetal pregnancy, and highest serum bile-acid concentration prior to randomisation (< 40 μmol/L, ≥ 40 μmol/L).

The minimisation algorithm will be generated by MedSciNet, which will hold the allocation code. Research teams at sites will approach women to confirm eligibility and the intervention will be allocated using the web-based randomisation to provide an alpha numeric pack number which will correlate with a pack to be dispensed by that site’s pharmacy. The trial is triple-masked: trial participants, clinical care providers, outcome assessors and data analysts will all be masked to allocation.

Emergency code break will be available, but clinicians requesting unmasking must be satisfied that it is a genuine emergency and that knowledge of the treatment allocation (either UDCA or placebo) is needed to guide the appropriate clinical management of the participant. In some cases this may be achieved without unblinding, by stopping the allocated treatment and treating the participant with UDCA.

### Analysis

The analysis and presentation of results will follow the most up-to-date recommendations of the Consolidated Standards of Reporting Trials (CONSORT) group. Full details of the Statistical Analysis Plan are given in Additional file 1: Text S1. Analyses will be completed in Stata® version 13.1 or later. Unmasked data will only be made available for analysis after full database lock (after all data outcomes have been completed) or on request by the Data Monitoring Committee. All analyses will follow the intention-to-treat principle, i.e. all randomised women (and infants) will be analysed according to the treatment that they were allocated to, irrespective of the treatment they received or whether they received any treatment at all.

Demographic and clinical data will be summarised with counts and percentages for categorical variables, means with standard deviations for normally distributed continuous variables, and medians with interquartile or simple ranges for other continuous variables. All comparative analyses will be performed adjusting for minimisation factors at randomisation [15]. Centre will be included as a random effect. Both unadjusted and adjusted effect estimates will be presented, but the primary inference will be based on the adjusted estimates.

Binary outcomes will be analysed using log binomial regression models. Results will be presented as adjusted risk ratios plus confidence intervals. If any model does not converge, a Poisson regression model with robust variance estimation will be used [16]. Continuous outcomes will be analysed using linear regression models and results will be presented as adjusted differences in means with confidence intervals. Continuous outcomes will not be categorised for statistical testing unless pre-specified as an outcome (and clinically relevant). Unadjusted median differences with confidence intervals will be presented for skewed continuous variables, and an adjusted analysis using quantile regression will be presented if possible. Analysis of outcomes that are measured repeatedly over time (severity of itch and biochemistry measures) will use repeated measures analysis techniques. Alternatively, if the data are highly skewed, geometric means of the post-randomisation observations will be reported [17] and the trial arms will be compared using a geometric mean ratio, adjusted for the baseline measures and minimisation factors.

The analysis of perinatal outcomes will include all infants born to a randomised mother, so the denominator will be the number of infants. For these outcomes, correlations between twins will be accounted for in the adjusted model by nesting twin cluster as a random effect within centre. Multiplicity (multi-fetal pregnancy) will also be adjusted for as a fixed effect in the models.

#### Pre-specified subgroup analysis

Pre-specified subgroup analyses will be performed for the primary outcome and its components, the bile-acid and itch outcomes, using the statistical test of interaction (or test for trend). Binary outcomes will be presented as risk ratios with confidence intervals on a forest plot. Pre-specified subgroups will be based on the criteria selected for minimisation: serum bile-acid concentration at baseline (10–39 μmol/L/ ≥ 40 μmol/L); gestational age (participants recruited before 34 weeks, 34 to 36 + 6 weeks, ≥ 37 weeks’ gestation); singletons and twins.

#### Sensitivity analyses

Sensitivity analyses will be conducted for the primary outcome, itch and bile-acid concentration between randomisation and delivery, excluding mothers or infants of mothers did not adhere to the intervention (< 90% medication adherence consistently self-reported).

#### Level of statistical significance

Ninety-five percent confidence intervals will be used for all primary and secondary outcome comparisons including subgroup analysis.

#### Missing data

Missing data as a result of women or infants being lost to follow-up are expected to be less than 5%. All complete data items collected will be used. Case note review will be conducted as usual on women who discontinue the intervention, and on women who withdraw if they indicated consent for outcome ascertainment.

### Economic analysis

Data on mother and infant inpatient care and mode of delivery will be costed using nationally published sources. The cost of UDCA (derived from *British National Formulary*, National Institute for Health and Care Excellence) will also be included for women randomised to receive the intervention. Descriptive statistics will be reported including mean cost per participant and 95% confidence intervals constructed using bootstrapping.

### Assessment of safety and reporting

At each clinic visit, a member of the clinical or research team will ask the woman if she has had any adverse events, and will ensure that she has appropriate clinical monitoring as routinely performed in each maternity unit. Standard definitions of an adverse event, adverse reaction, serious adverse event, serious adverse reaction and suspected unexpected serious adverse reaction will be followed. The relationship of each adverse event to the IMP will be determined by a medically qualified individual according to the usual definitions of causality. The period for safety reporting will be from first dose of IMP until discharge of mother and discharge of infant. Expectedness will be determined according to the Summary of Product Characteristics for UDCA. Standard reporting guidelines will be followed.

The following expected adverse events and serious adverse events are considered to be expected in this population of pregnant women or a result of the routine care/treatment of a participant and as such do not need to be recorded:

- Worsening pruritus
- Admission in active labour
- Admission for cervical ripening or induction of labour
- Admission for caesarean section
- Admission for assessment for suspected fetal compromise, including poor growth, or reduced fetal movements
- Admission for monitoring for hypertension, antepartum haemorrhage, suspected preterm labour, pre-labour rupture of the membranes or other reasons for monitoring
- Admission for psychiatric or social reasons
- Admission for unstable lie or external cephalic version.

The following fetal and neonatal outcomes are pre-specified outcomes and as such will be recorded on electronic Case Record Forms but not expeditiously reported:

- Neonatal unit admission
- Stillbirth and perinatal death (within 7 days)
- Preterm delivery (< 37 completed weeks’ gestation)
- Meconium staining of the amniotic fluid or placenta
- Small for gestational age
- Seizures
- Encephalopathy (treated with hypothermia)
- Need for respiratory support – ventilation via an endotracheal tube (ETT) or nasal continuous positive airway pressure (CPAP)
- Sepsis requiring antibiotics with symptoms or confirmed blood or cerebrospinal fluid (CSF) culture

An unexpected serious adverse event is any event that meets the definition of a serious adverse event and is not detailed in the list above as expected, including:

- Maternal death
- Maternal acute hepatic failure resulting in admission to an intensive care setting or requiring liver transplant
- Any unexpected fetal or perinatal death unrelated to ICP

### Quality control and assurance

Initiation of each participating centre will be performed by the chief investigator or their delegate once all appropriate approvals are in place and the IMP has been shipped to the site. During the trial, ongoing on-site and central monitoring will be conducted.

The site principal investigator (PI), research midwife and their delegates from each recruiting centre will be fully trained in protocol adherence and able to deal with site-specific issues. They will then be responsible for delivering this training to all relevant site staff prior to opening their centre for recruitment. The PI and research midwife will also promote the trial, and ensure that all appropriate site staff, are kept fully appraised of issues such as recruitment status, informed consent, data collection, follow-up and changing regulations, so that the necessary recruitment targets are reached within the timescale.

The trial coordinator will monitor recruitment against targets, and monitor data collection completeness and quality on a day-to-day basis.

Throughout the trial, there will be central monitoring, overseen by the Project Management Group, Data Monitoring Committee, Trial Steering Committee and Quality Assurance Team, ensuring good communication between National Perinatal Epidemiology Unit trial team and the site staff. Trial monitoring will be conducted in accordance with the monitoring plan developed from the trial-specific risk assessment. The monitor will visit sites where anomalies are identified through central monitoring. Sites that are identified as requiring additional support will be visited by a member of the trial team or the monitor as appropriate to the particular issues.

The Data Monitoring Committee will look regularly at protocol adherence by site and by trial arm, including randomisation processes and patterns of allocation.

#### Participant confidentiality, data handling and record keeping

Data will be entered by trained researchers at site onto a web-based database with pre-specified sense checks and boundary checks. Data will be downloaded and stored securely at the Clinical Trials Unit on a monthly basis as a minimum. Data will be reviewed by midwife coordinators and queries raised where clarification is needed. Source data will be verified by a data monitor in 5% of records by comparison of source data against electronic data entry. Data management procedures will be carried out in accordance with National Perinatal Epidemiology Unit Clinical Trials Unit’s Standard Operating Procedures and the principles outlined within MedSciNet Clinical Trial Framework User Manual.

All paper documents will be stored securely and kept in strict confidence in compliance with the Data Protection Act (1998) and all trial data will be stored in line with the Medicines for Human Use (Clinical Trials) Amended Regulations 2006. Due to the nature of pregnancy research, data will be kept for a period of no fewer than 25 years in order to follow up on health-related issues which may become relevant in the future. At all times the personal data will be held securely and will not be used for any other purpose.

The dataset will be available to appropriate academic parties on request from the chief investigator in accordance with the data-sharing policies of King’s College London and the National Perinatal Epidemiology Unit Clinical Trials Unit, with input from the Co-investigator Group where applicable.

## Discussion

Current practice in the UK at the time of trial commencement for treatment of ICP is inconsistent, with some units routinely prescribing UDCA, others prescribing very little and the remainder offering it variably. We have engaged with all the sites to assess readiness and willingness to participate for the duration of the trial and offer women this placebo-controlled trial. We have also worked for many years with the patient support group, ICP Support, to ensure that we are providing accurate, up-to-date information on treatment options for women with ICP. Our previous pilot trial of UDCA in women with ICP demonstrated that the trial would be feasible and the research question remains active and unanswered. Results are highly likely to influence clinical practice, through direct management and impact on national and international guidelines.

### Trial status

Protocol: Version 3.1: 5 April 2018. Final approval: 31 July 2015. Trial opened to recruitment: 23 October 2015. First participant recruited: 23 December 2015. All 33 sites (30 NHS trusts) opened: 7 December 2016. End of recruitment: 31 August 2018.

## Additional files

Additional file 1: **Text S1.** Statistical Analysis Plan. (PDF 701 kb)

Additional file 2: **Appendix 1.** Participant information leaflet. (PDF 1717 kb)

Additional file 3: **Appendix 2.** Consent form. (PDF 366 kb)

## Abbreviations

ICP: Intrahepatic cholestasis of pregnancy
IMP: Investigational Medicinal Product
UDCA: Ursodeoxycholic acid
