# Supplementary material for: Ursodeoxycholic acid versus placebo in the treatment of women with intrahepatic cholestasis of pregnancy (ICP) to improve perinatal outcomes: protocol for a randomised controlled trial (PITCHES)
Source: Trials. 2018 Nov 27;19:657. doi: 10.1186/s13063-018-3018-4 (PMC6260710; doi:10.1186/s13063-018-3018-4)
Supplement: Supplementary file 1 — Text S1. Statistical Analysis Plan. (PDF 701 kb) [file 13063_2018_3018_MOESM1_ESM.pdf]

PITCHES

Phase III trial in IntrahepaTic CHolestasis of pregnancy (ICP) to Evaluate urSodeoxycholic acid (UDCA) in improving perinatal outcomes

Statistical Analysis Plan
Version 1.2
Date: 10 August 2018

Author: *Jennifer Bell (Trial Statistician, NPEU CTU)*
Reviewers: *Pollyanna Hardy*
(Senior Statistician, 2015–2017, NPEU CTU)
Dr Louise Linsell
(Senior Statistician, 2017–present, NPEU CTU)
Professor Lucy Chappell
(Chief Investigator, St Thomas' Hospital)

Protocol version: 3.2

CONTENTS

List of abbreviations....4
1 Introduction....5
2 Background information....6
2.1 Rationale....6
2.2 Objectives of the trial....6
2.3 Trial design....7
2.4 Eligibility....7
2.5 Interventions....7
2.6 Definition of primary and secondary outcomes....7
2.6.1 Primary outcomes....8
2.6.2 Secondary outcomes....8
2.7 Hypothesis framework....9
2.8 Sample size & power....9
2.9 Intervention allocation....10
2.10 Data collection schedule....10
2.11 Interim analyses and stopping rules....11
2.11.1 Trial stopping rules....12
2.12 Trial reporting....12
3 Protocol non-compliances....12
3.1 Major....12
3.2 Minor....13
4 Adherence to the intervention....13
5 Analysis populations....14
5.1 Post-randomisation exclusions....14
5.2 Population definitions....14
5.2.1 Descriptive analysis population....14
5.2.2 Comparative analysis population....14
5.2.3 Interim analysis population....14
6 Descriptive analyses....15
6.1 Representativeness of trial population and participant throughput....15
6.2 Baseline comparability of randomised groups....15
6.3 Losses to follow-up....16
7 Comparative analyses....16
7.1 Detailed definition of outcomes....16

7.2 Primary analysis 16
7.3 Secondary analyses 17
7.4 Pre-specified subgroup analyses 17
7.5 Sensitivity analyses 18
7.6 Significance levels 18
7.7 Missing data 18
7.8 Statistical software 18
8 Safety data analysis 18
9 Additional exploratory analysis 19
10 Deviation from analysis described in protocol 19
11 References 20
11.1 Trial documents 20
11.2 Other references 20
12 Approval 21
13 Document history 22

LIST OF TABLES

Table 1: Trial assessments 11

LIST OF ABBREVIATIONS

AE	Adverse event
ALT	Alanine transaminase
ANCOVA	Analysis of covariance
AST	Aspartate aminotransferase
CI	Confidence interval
CONSORT	Consolidated Standards of Reporting Trials
CPAP	Continuous positive airway pressure
CTG	Cardiotocography
CTU	Clinical Trials Unit
eCRF	Electronic case report form
EEG	Electroencephalography
EME	Efficacy Mechanisms & Evaluation
g	Gram
ICP	Intrahepatic Cholestasis of Pregnancy
ITT	Intention to treat
IUGR	Intrauterine growth restriction
IVH	Intraventricular haemorrhage
kg	Kilogram
L	Litre
mg	Milligrams
NIHR	National Institute for Health Research
NNU	Neonatal unit
NPEU	National Perinatal Epidemiology Unit
OC	Obstetric cholestasis
pH	Power of hydrogen
PI	Principal investigator
RR	Risk ratio
SAE	Serious adverse event
SAP	Statistical analysis plan
SUSAR	Suspected Unexpected Serious Adverse Reactions
UDCA	Ursodeoxycholic acid
µmol	Micromole

1 INTRODUCTION

This document details the proposed presentation and analysis for the main paper(s) reporting results from the National Institute for Health Research (NIHR) Efficacy Mechanisms & Evaluation (EME) programme funded multi-centre, masked, randomised controlled trial PITCHES.

The results reported in these papers will follow the strategy set out here. Subsequent analyses of a more exploratory nature will not be bound by this strategy, though they are expected to follow the broad principles laid down here. The principles are not intended to curtail exploratory analysis (example: to decide cut-points for categorisation of continuous variables), nor to prohibit accepted practices (example: data transformation prior to analysis), but they are intended to establish the rules that will be followed, as closely as possible, when analysing and reporting the trial.

The analysis plan will be available on request when the principal papers are submitted for publication in a journal. Suggestions for subsequent analyses by journal editors or referees, will be considered carefully, and carried out as far as possible in line with the principles of this analysis plan; if reported, the source of the suggestion will be acknowledged.

Any deviations from the statistical analysis plan will be described and justified in the final report of the trial. The analysis should be carried out by an identified, appropriately qualified and experienced statistician, who should ensure the integrity of the data during their processing. Examples of such procedures include quality control and evaluation procedures.

2 BACKGROUND INFORMATION

2.1 Rationale

Intrahepatic cholestasis of pregnancy (ICP), also called obstetric cholestasis (OC), is the most common liver disorder specific to pregnancy. It presents with maternal pruritus, raised concentrations of serum bile acids and abnormal liver function tests. The maternal symptoms typically resolve postpartum, but affected women have an increased risk of hepatobiliary disease in later life (Marschall et al., 2013). ICP is associated with increased rates of spontaneous and iatrogenic preterm labour, fetal hypoxia, meconium-stained amniotic fluid (Glantz et al., 2004, Chappell et al., 2012, Geenes and Williamson, 2009). There are also reports of increased rates of intrauterine death (Fisk et al., 1998, Davies et al., 1995, Williamson et al., 2004), although the incidence is low (Glantz et al., 2004, Geenes et al., 2014). Most clinicians treat ICP with ursodeoxycholic acid (UDCA) (Saleh et al., 2007, Zapata et al., 2005) to improve maternal pruritus and biochemical abnormalities.

However, there are currently no data to support the use of UDCA to improve pregnancy outcome as none of the trials performed to date have been powered to address this question.

UDCA is a naturally occurring bile acid that is present in small amounts in humans. It is relatively hydrophilic and has several actions that result in improvement of cholestasis. The main clinical research question is whether adverse pregnancy outcomes can be reduced in women with ICP by treatment with UDCA.

The recent PITCH pilot trial in 111 ICP women demonstrated that UDCA decreased maternal itching compared to placebo but by less than the difference pre-specified as clinically meaningful (Chappell et al., 2012).

The latest updated Cochrane review (Gurung et al., 2013) judged many of the primary trials to be at moderate to high risk of bias. Trials to date have lacked power to demonstrate whether UDCA is fetoprotective, with numbers of participants and adverse events too small to enable recommendation of UDCA. The Cochrane review concluded that larger trials of UDCA to determine fetal benefits or risks are needed.

If UDCA is found to be beneficial in ameliorating adverse perinatal outcomes, once published these results would be highly likely to lead to an immediate change in clinical practice, through individual choice of clinicians and women, and through changing national/international guidelines.

2.2 Objectives of the trial

The primary short term objective of the trial is to determine if UDCA treatment of women with ICP between 20^{+0} and 40^{+6} weeks of gestation reduces the following adverse perinatal outcomes up to infant hospital discharge:

- In utero fetal death after randomisation
- Known neonatal death up to 7 days
- Preterm delivery (less than 37 weeks' gestation)
- Neonatal unit admission for at least 4 hours.

The secondary objectives of the trial are:

- To investigate the effect of UDCA on other short term outcomes for both mother and infant
- To assess the impact of UDCA on health care resource use: in terms of the total number of nights for mother and infant, together with level of care.

2.3 Trial design

This will be a masked placebo-controlled randomised trial, to evaluate UDCA vs. placebo in women with ICP between 20^{+0} and 40^{+6} weeks' gestation. The study will be conducted with 580 women at approximately 30 centres across the UK.

It is anticipated that the trial will last four years. Recruitment will run for approximately 39 months.

Recruitment will be rolled out to centres, with a staggered start. Following recruitment of the final participant we will allow six months for completion of pregnancy of all remaining participants and for their infants to be discharged home. This will be followed by data cleaning and analysis.

2.4 Eligibility

<u>Inclusion criteria:</u>

- ICP (pruritus with a raised serum bile acid above the upper limit of normal for the local laboratory),
- 20^{+0} to 40^{+6} weeks' gestation on day of randomisation,
- No known lethal fetal anomaly,
- Singleton or twin pregnancy,
- Aged 18 years or over,
- Able to give written informed consent.

<u>Exclusion criteria:</u>

- Decision already made for delivery within the next 48 hours,
- Known allergy to any component of the UDCA or placebo tablets,
- Triplet or higher-order multiple pregnancy.

2.5 Interventions

<u>Treatment group:</u>

UDCA 1 g daily (500 mg bd), increased in increments of 500 mg per day every 3–14 days if there is no biochemical or clinical improvement, based on clinical decision, to a maximum of 2 g per day. The dose of IMP may be reduced to 500 mg daily.

Administered orally as Ursofalk tablets each containing 500 mg UDCA.

<u>Comparator:</u>

Identical placebo tablets administered in the same dose increments orally.

2.6 Definition of primary and secondary outcomes

2.6.1 Primary outcomes

Primary perinatal outcomes:
A composite of perinatal death (as defined by in utero fetal death after randomisation or known neonatal death up to 7 days) or preterm delivery (less than 37 weeks' gestation) or neonatal unit admission for at least 4 hours (between infant delivery and hospital discharge).

Each infant will only be counted once within this composite.

2.6.2 Secondary outcomes

Secondary maternal outcomes:
- Maternal serum concentration (between randomisation and delivery) of the following biochemical indices of disease:
  - Bile acids
  - Alanine transaminase
  - Aspartate transaminase
  - Bilirubin (total)
  - Gamma glutamyl transferase
- Itch between randomisation and delivery, as measured by the worst episode of itch over past 24 hours (mm on visual analogue scale, assessed at clinic visits)
- Maximum dose of trial medication required
- Need for additional therapy for cholestasis
- Gestational diabetes mellitus
- Assessment of myometrial contractions by CTG approximately one week (3–14 days) post randomisation
- Mode of onset of labour
- Reason for induction or pre-labour caesarean section
- Estimated blood loss after delivery
- Maternal death.

Secondary perinatal outcomes:
- In utero fetal death after randomisation
- Preterm delivery (less than 37 weeks' gestation)
- Known neonatal death up to 7 days
- Known neonatal death up to 28 days
- NNU admission for at least 4 hours
- Mode of delivery classified as spontaneous vaginal, instrumental vaginal or caesarean
- Number of nights in each category of care (intensive, high dependency, special, transitional and normal)
- Total number of nights in neonatal unit
- Birth weight (g)
- Birth weight centile
- Gestational age at delivery
- Presence of meconium
- APGAR score at 5 minutes

- Umbilical arterial pH at birth
- Need for supplementary oxygen prior to discharge
- Number of days when supplemental oxygen is required
- Need for ventilation support (CPAP/high flow/endotracheal ventilation)
- Abnormal cerebral ultrasound scan
- Confirmed sepsis (positive blood or cerebrospinal fluid cultures)
- Necrotising Enterocolitis (Bell's stage 2 and 3)
- Seizures (confirmed by EEG or requiring anticonvulsant therapy)
- Encephalopathy (treated with hypothermia)
- Other indications and main diagnoses resulting in neonatal unit admission for at least 4 hours.

2.7 Hypothesis framework

This is a superiority trial, comparing UDCA with placebo. Analysis of the trial will entail calculation of treatment effect measures and confidence intervals to assess the difference between the two arms.

2.8 Sample size & power

We aim to recruit 580 women in total; this will allow for the possibility of 5% of infants being lost to follow-up and is a conservative estimate given that some women will have twin pregnancies.

The primary outcome measure will be a composite of perinatal death or preterm delivery (less than 37 weeks' gestation) or NNU admission. The sample size is informed by the most recent Cochrane meta-analysis (Gurung et al., 2013). This includes the trials reported in the previous meta-analysis (Bacq et al., 2012) with the addition of the largest trial published in 2012 by our group (Chappell et al., 2012).

From these data, we can estimate the event rate for infants of untreated women as 40% with a plausible and relevant reduction to 27% for infants of women treated with UDCA, corresponding to an absolute risk reduction of 13% and a risk ratio (RR) of 0.675. This is conservative compared with the effect sizes seen in the Cochrane meta-analysis (Gurung et al., 2013) for the three individual endpoints (RR 0.31, 0.46 and 0.48 for perinatal death, preterm delivery and NNU admission respectively). 550 infants of women with ICP (275 per group) are required to have a 90% chance of detecting, as significant at the 2-sided 5% level, a reduction in the primary outcome measure from 40% in the control group to 27% in the treated group. Allowing for 5% being lost to follow-up requires a total sample size of 580 infants (290 per group). We are uncertain as to the proportion of women that have twin pregnancies in this target population. Office for National Statistics data indicate that around 1.5% of mothers have twin deliveries (ONS Birth Statistics, 2008). Recruiting 580 women to achieve a sample size of 580 infants is, therefore, a conservative estimate of the sample size required to address the primary (short term) objective.

This number will also allow us to look at the components of the composite endpoints: a trial assessing 550 infants will have 89% power to demonstrate a reduction in NNU admission rates from 17% to 8%, and 99% power for a reduction in prematurity from 41% to 23% (based on the Cochrane meta-analysis – Gurung et al., 2013), both effect sizes of the same

magnitude as that demonstrated in the previous PITCH trial (Chappell et al., 2012). We do not anticipate enough perinatal deaths to detect reliably any plausible treatment effect but we have included this due to its clinical importance and will report it separately.

If there is sufficient time within the existing study time-line, additional participants will be recruited up to the number of women who discontinued the intervention or withdrew.

2.9 Intervention allocation

The allocation ratio of intervention (ursodeoxycholic acid) to control (placebo) arms will be 1:1. Randomisation will be managed via a secure web-based randomisation facility hosted by MedSciNet with telephone back-up available. A minimisation algorithm will be used to ensure balance between the groups with respect to study centre (approximately 30 centres), gestational age at randomisation (< 34, 34 to < 37, ≥ 37 weeks' gestation), single vs. multi-fetal pregnancy, and serum bile acid concentration prior to randomisation (< 40 µmol/L, ≥ 40 µmol/L). MedSciNet will hold the allocation code.

2.10 Data collection schedule

Clinical information will be collected using the following case report forms (CRFs) in a MedSciNet database:

- Screening Log
- Prior to Randomisation
- Randomisation
- Maternal Details
- Contact Details
- Study Product
- Antenatal Visit
- Maternal Delivery
- Infant Delivery
- Maternal Discharge
- Infant Discharge
- Adverse Events (Mother)
- Serious Adverse Events
- Discontinuation of Intervention
- Trial Withdrawal.

See Table 1 for a schedule of trial assessments.

Table 1: Trial assessments

Procedure	Screening[1]	Trial Entry and treatment	Weekly + four days[2]	Delivery	At hospital discharge to home
Demography		✓			✓
Confirmation of Eligibility	✓				
Consent[3]		✓			
Randomisation		✓			
Blood samples for bile acids/ALT/[4]	✓	✓	✓		
IMP dosing[5]		✓	✓		
CTG[6]			(✓) (first visit after randomisation only)		
Pruritus score on itching chart		(✓)	(✓)		
SAEs[7]		✓	✓	✓	✓
Concomitant Medication[8]		✓	✓	✓	✓
Tablet adherence assessment			✓		
Post-delivery outcome form					✓

1. All screening assessments are part of routine clinical practice.
2. Weekly visits are recommended but not mandatory; normal hospital clinical practice is acceptable.
3. No trial specific procedures before consent.
4. These blood tests are taken as per routine clinical practice and are not trial specific.
5. IMP started after randomisation. IMP dose altered by PI if indicated by symptoms and/or blood tests taken during normal clinical practice.
6. CTG only measured 1 week after randomisation or as per routine clinical practice.
7. All unexpected AEs occurring during the trial that are observed by the PI or reported by the participant will be recorded in the eCRF, whether or not attributed to the IMP. Unexpected SAEs will be expeditiously reported.
8. All prescribed concomitant medication only.

2.11 Interim analyses and stopping rules

A Data Monitoring Committee (DMC), independent of the trial organisers, has been established to ensure the wellbeing of study participants and will review the trial's progress. Interim analyses will be supplied, in strict confidence, to the DMC as frequently as its Chair requests. The terms of reference for the DMC were agreed at their first meeting, and a DMC

Charter was completed and signed by all members. Meetings of the committee will be arranged annually, or more often as appropriate.

The trial statistician will produce (or oversee the production of) reports for the DMC and will participate in DMC meetings, guiding the DMC through reports, and will also take meeting minutes. A template report/dummy tables will be agreed.

The DMC is the only body involved in the trial that has access to the unblinded comparative data. It will receive and review the progress and accruing data of the trial and provide advice on the conduct of the trial to the Trial Steering Committee (TSC). The trial statistician will produce the report unblinded to allocation.

Unless modification or cessation of the trial is recommended by the DMC, the TSC, investigators, collaborators and administrative staff (except those who supply the confidential information) will remain ignorant of the results of the interim analysis. Collaborators and all others associated with the study may write to the DMC via NPEU CTU, to draw attention to any concern they may have about the possibility of harm arising from the treatment under study. See the DMC Charter for more information.

2.11.1 Trial stopping rules

The DMC will periodically review study progress and outcomes as well as reports of unexpected SAEs. In the light of interim data and other evidence from relevant studies, the DMC will inform the TSC if, in its view, there is proof beyond reasonable doubt that the data indicate that the trial should be terminated. A decision to inform the TSC of such a finding will in part be based on statistical considerations. Appropriate proof beyond reasonable doubt cannot be specified precisely. A difference of at least 3 standard errors in the interim analysis of a major endpoint may be needed to justify halting or modifying the trial prematurely, for the superiority hypothesis.

2.12 Trial reporting

The trial will be reported according to the principles of the CONSORT statement. The final analysis will be conducted for all outcomes collectively, at the end of the trial.

3 PROTOCOL NON-COMPLIANCES

A protocol non-compliance is defined as a failure to adhere to the protocol such as the wrong intervention being administered, incorrect data being collected and documented, errors in applying inclusion/exclusion criteria or missed follow-up visits due to error.

All protocol non-compliances will be summarised by trial arm in the final report.

3.1 Major

Data considered fraudulent is the only pre-defined major protocol non-compliances with a direct bearing on the primary outcome.

3.2 Minor

The following are defined as minor protocol non-compliances:

Participants randomised in error:

These include participants:

- Who do not have ICP (pruritus with a raised serum bile acid above the upper limit of normal for the local laboratory)
- Who are < 20^{+0} or > 40^{+6} weeks' gestation on day of randomisation
- With a known lethal fetal anomaly
- With triplets or higher-order multiple pregnancy
- Aged under 18 years
- Whose consent to take part has not been documented
- Where a decision to deliver within the next 48 hours has been made
- With a known allergy to any component of the UDCA or placebo tablets.

Participants who do not receive allocated intervention:

These include participants:

- in the UDCA arm who did not receive UDCA (i.e. received the placebo or no study treatment)
- in the placebo arm who did not receive the placebo (i.e. received UDCA or no study treatment).

Deviations related to treatment:

- Participants who were given UDCA in addition to the allocated treatment (i.e. received open-label UDCA, in addition to the dosing regime defined in the protocol).

4 ADHERENCE TO THE INTERVENTION

At each antenatal follow-up visit with a member of the research team, women will be asked the percentage of IMP that they have taken since their last appointment and this will be recorded. No IMP accountability is required for this trial. However, if a participant does return their medication, the research team will be responsible for returning any participant's IMP to pharmacy for reconciliation and verification prior to on-site destruction.

A sensitivity analysis will be performed for the primary outcome, itch, and bile acid between randomisation and delivery, on women or babies of women who have adhered, having consistently (as reported at each antenatal visit) taken ≥ 90% of the intervention, compared with those who have not (< 90% of the intervention). It should be noted that this information is self-reported.

See also section 7.5.

5 ANALYSIS POPULATIONS

Women and infants will be analysed in the groups to which the women were randomly assigned, comparing the outcome of all in the UDCA arm with all in the placebo arm, regardless of deviation from the protocol or treatment received (referred to as the Intention to Treat (ITT) population).

The unit of randomisation is the mother, which raises the issue of non-independence of observations if the mother gives birth to twins. For perinatal outcomes, the denominator will be the number of infants. For these outcomes, correlations between twins will be accounted for in the adjusted model.

5.1 Post-randomisation exclusions

The following will be excluded from the analysis populations post-randomisation:

- Participants for whom full consent was not obtained

- Participants for whom consent to use their data was withdrawn (women can specify whether data collected up to the point of withdrawal can be used. If the response is ‘No’, then they will be considered post-randomisation exclusions. If the response is ‘Yes’, then they will be reported as ‘missing’ for any data not collected after withdrawal)

- Participants for whom fraudulent data was detected (should fraudulent data be detected, consideration will be given to excluding all data for the site where such data were found).

The numbers (with percentages of the randomised population) of post-randomisation exclusions will be reported by trial arm, and reasons summarised, in the CONSORT flow diagram.

5.2 Population definitions

5.2.1 Descriptive analysis population

Baseline demographic and clinical characteristics will be reported for all women randomised for whom data are available, excluding post-randomisation exclusions.

5.2.2 Comparative analysis population

Maternal outcomes

All women randomised, excluding post-randomisation exclusions.

Perinatal outcomes

All infants born to a randomised mother, the denominator being the total number of infants. This will also exclude post-randomisation exclusions.

5.2.3 Interim analysis population

Different denominators will be used in the interim analysis:

- Baseline data will be reported for all trial participants with available data, excluding

known post-randomisation exclusions.

- Outcome data will be reported for participants who can be described as 'completers' (i.e. all trial participants with available data who have delivered, died, been transferred or discharged) at the time of the database snapshot, excluding known post-randomisation exclusions.
- Safety data will be reported for all trial participants with available data, excluding known post-randomisation exclusions.

6 DESCRIPTIVE ANALYSES

6.1 Representativeness of trial population and participant throughput

The flow of participants through each stage of the trial will be summarised using a CONSORT diagram. We will report the numbers of participants:

- Assessed for eligibility
- Excluded (not eligible)
- Eligible
- Excluded (eligible) (with reasons)
- Randomised
- Randomised in error
- Allocated to each intervention
    - Received allocated intervention
    - Discontinued intervention
    - Withdrew consent to use data
- Included in the analysis
- Excluded from analysis
    - Withdrew consent to continue to collect outcome data.

6.2 Baseline comparability of randomised groups

Participants in the two randomised groups will be described separately with respect to their demographic and clinical characteristics at trial entry.

Numbers (with percentages) for binary and categorical variables and means (and standard deviations), or medians (with interquartile range, and minimum and maximum values if appropriate) if the data are skewed, for continuous variables will be presented. There will be no tests of statistical significance performed nor confidence intervals calculated for differences between randomised groups on any baseline variable.

This will include the following information:

- Maternal demographic and pregnancy characteristics at trial entry
- Previous obstetric and medical history
- Maternal clinical characteristics at randomisation.

6.3 Losses to follow-up

The number (with percentages) of losses to follow-up among infants will be reported for the two trial arms, and the reasons will be recorded. Any deaths (and their causes) will be reported separately.

7 COMPARATIVE ANALYSES

7.1 Detailed definition of outcomes

Derivations of the outcomes are described in the data derivation spreadsheet.

7.2 Primary analysis

The placebo group will be used as the reference group in all analyses. All comparative analyses will be performed adjusting for minimisation criteria: centre (approximately 30 centres), gestational age at randomisation (< 34, 34 to < 37, ≥ 37 weeks' gestation), single vs. multi-fetal pregnancy, and serum bile acid concentration prior to randomisation (< 40 µmol/L, ≥ 40 µmol/L). Centre will be included as a random effect. Both unadjusted and adjusted effect estimates will be presented, but the primary inference will be based on the adjusted estimates.

All binary outcomes will be analysed using a log binomial regression model. The results will be presented as adjusted risk ratios with confidence intervals. If any model does not converge, a Poisson regression model with robust variance estimation will be used (Zou, 2004). Continuous outcomes will be analysed using linear regression models and results will be presented as adjusted differences in means with confidence intervals. Unadjusted median differences with confidence intervals will be presented for skewed continuous variables, and an adjusted analysis using quantile regression will be presented if possible.

The analysis of perinatal outcomes will include all infants born to a randomised mother, so the denominator will be the number of infants. Correlations between twins will be accounted for in the adjusted model by nesting twin cluster as a random effect within centre. Multiplicity will also be adjusted for as a fixed effect in the models since it is also a minimisation criterion.

The following secondary outcomes will be described only and no formal statistical analysis comparing groups will be conducted:

<u>Secondary maternal outcomes</u>

- Maximum dose of trial medication required
- Need for additional therapy for cholestasis
- Assessment of myometrial contractions by CTG approximately one week (3–14 days) post randomisation
- Reason for induction or pre-labour caesarean section
- Maternal death.

<u>Secondary perinatal outcomes</u>

- Known neonatal death up to 28 days
- Number of nights in each category of care (intensive, high dependency, special, transitional and normal)
- Need for supplementary oxygen prior to discharge
- Number of days when supplemental oxygen is required
- Need for ventilation support (CPAP/high flow/endotracheal ventilation)
- Abnormal cerebral ultrasound scan
- Confirmed sepsis (positive blood or cerebrospinal fluid cultures)
- Necrotising Enterocolitis (Bell's stage 2 and 3)
- Seizures (confirmed by EEG or requiring anticonvulsant therapy)
- Encephalopathy (treated with hypothermia)
- Other indications and main diagnoses resulting in neonatal unit admission for at least 4 hours.

Repeated measures

Severity of itch, bile acid and alanine transaminase serum concentrations are measured at baseline prior to randomisation and then repeatedly over time at antenatal visits post-randomisation. For these outcomes, the null hypothesis is that there is no difference in change between the allocation groups. These visits do not take place at fixed time points and hence participants will have a varying number of observations captured, so the data will be unbalanced. The data will be inspected for normality and heteroscedasticity and the most appropriate analysis will be selected depending upon the number of antenatal visits per woman and the distribution of the outcomes. In addition, the measures will be plotted over gestational age, and time since randomisation.

One option is to use a repeated measures ANCOVA to take account of the within-subject correlation between measures at the post-randomisation antenatal visits. Baseline measures at randomisation and minimisation factors will be adjusted for. The repeated measures p-value will be reported.

Alternatively if the data do not meet the assumptions of the ANCOVA model, geometric means of the post-randomisation observations will be reported (Matthews et al., 1990). The trial arms will be compared using a geometric mean ratio, adjusted for the baseline measures and minimisation factors.

7.3 Secondary analyses

No secondary analyses have been planned.

7.4 Pre-specified subgroup analyses

Pre-specified subgroup analyses will be performed for the primary outcome and its components, the bile acid and itch outcomes, using the statistical test of interaction. Binary outcomes will be presented as risk ratios with confidence intervals on a forest plot.

Pre-specified subgroups will be based on the criteria used for minimisation:

- Serum bile acid concentrations at baseline (10–< 40 μmol/L/ ≥ 40 μmol/L)
- Gestational age (participants recruited at < 34 weeks, 34 to < 37 weeks, ≥ 37 weeks' gestation)
- Singletons and twins.

7.5 Sensitivity analyses

Sensitivity analyses will be conducted for the primary outcome, itch and bile acid between randomisation and delivery, excluding mothers or infants of mothers who did not adhere to the intervention (< 90% consistently self-reported).

7.6 Significance levels

95% confidence intervals will be used for all primary and secondary outcome comparisons, including all subgroup analyses.

7.7 Missing data

Missing data will be described by presenting the number of individuals in the missing category. All complete data collected on data collection forms will be used, since only essential data items will be collected.

7.8 Statistical software

Analyses will be completed in Stata® version 13.1 or later.

8 SAFETY DATA ANALYSIS

Serious Adverse Events (SAEs)

SAEs will be summarised by trial arm, with numbers of each severity (mild, moderate or severe); causality (unrelated, possibly, probably, definitely related); and action taken (to be categorised when data is available).

Events will also be listed, reporting allocation, severity, relatedness to study drug, outcome, and details.

Suspected Unexpected Serious Adverse Reactions (SUSARs)

The number of SUSARs will be reported by trial arm, with causality (unrelated, possibly, probably, definitely related).

Adverse Events (AEs)

AEs will be summarised by trial arm, reporting numbers of each intensity (mild, moderate, or severe); relatedness to study drug; and outcome (persistent/ongoing, resolved, resolve with sequelae or death).

Events will also be listed, reporting allocation, intensity, causality, action taken, and details.

9 ADDITIONAL EXPLORATORY ANALYSIS

Any analyses not specified in the analysis protocol will be exploratory in nature and a 2-sided significance level of 0.01 will be used with 99% confidence intervals.

10 DEVIATION FROM ANALYSIS DESCRIBED IN PROTOCOL

None yet.

11 REFERENCES

11.1 Trial documents

SOP ST 105 v3.0 Statistical Analysis Plan
SOP ST 107 v2.0 Statistical Analysis and Reporting
PITCHES Trial Protocol version 3.2
PITCHES Dummy tables
PITCHES Data derivation

11.2 Other references

Chappell LC, Gurung V, Seed PT, Chambers J, Williamson C, Thornton JG. Ursodeoxycholic acid versus placebo, and early term delivery versus expectant management, in women with intrahepatic cholestasis of pregnancy: semifactorial randomised clinical trial. *BMJ* 2012; 344:e3799.

Davies MH, da Silva RC, Jones SR, Weaver JB, Elias E. Fetal mortality associated with cholestasis of pregnancy and the potential benefit of therapy with ursodeoxycholic acid. *Gut* 1995;37(4):580-4.

Fisk NM, Storey GN. Fetal outcome in obstetric cholestasis. *BJOG* 1988;95(11):1137-43.

Glantz A, Marschall HU, Mattsson LA. Intrahepatic cholestasis of pregnancy: Relationships between bile acid levels and fetal complication rates. *Hepatology* 2004;40:467-74.

Geenes V, Williamson C. Intrahepatic cholestasis of pregnancy. *World J Gastroenterol* 2009;15(17):2049-66.

Geenes V, Chappell LC, Seed PT, Steer PJ, Knight M, Williamson C. Association of severe intrahepatic cholestasis of pregnancy with adverse pregnancy outcomes: A prospective population-based case-control study. *Hepatology* 2014;59(4):1482-91.

Glantz A, Marschall HU, Mattsson LA. Intrahepatic cholestasis of pregnancy: Relationships between bile acid levels and fetal complication rates. *Hepatology* 2004;40:467-74.

Gurung V, Middleton P, Milan SJ, Hague W, Thornton JG. Interventions for treating cholestasis in pregnancy. *Cochrane Database Syst Rev*. 2013;24;6:CD000493.

Marschall HU, Wikström Shemer E, Ludvigsson JF, Stephansson O. Intrahepatic cholestasis of pregnancy and associated hepatobiliary disease: A population-based cohort study. *Hepatology* 2013;58(4):1385-9.

Matthews JN, Altman DG, Campbell MJ, Royston P. Analysis of serial measurements in medical research. *BMJ*. 1990 Jan 27;300(6719):230-5.

Saleh MM, Abdo KR. Consensus on the management of obstetric cholestasis: National UK survey. *BJOG* 2007;114(1):99-103.

Williamson C, Hems LM, Goulis DG, Walker I, Chambers J, Donaldson O, et al. Clinical outcome in a series of cases of obstetric cholestasis identified via a participant support group. *BJOG* 2004; 111(7):676-81.

Zapata R, Sandoval L, Palma J, Hernandez I, Ribalta J, Reyes H, et al. Ursodeoxycholic acid in the treatment of intrahepatic cholestasis of pregnancy. A 12-year experience. *Liver*

international: official journal of the International Association for the Study of the Liver 2005; 25(3):548-54.

Zou G. A modified Poisson regression approach to prospective studies with binary data. *Am. J Epidemiol.* 2004;159(7):702-6.

12 APPROVAL

<table>
<tr><td rowspan="2">Senior Statistician</td><td colspan="2">Name: Dr Louise Linsell</td></tr>
<tr><td>Signature</td><td>Date</td></tr>
<tr><td rowspan="2">Chief Investigator</td><td colspan="2">Name: Professor Lucy Chappell</td></tr>
<tr><td>Signature</td><td>Date</td></tr>
<tr><td rowspan="2">Chair of Trial Steering Committee (or delegate)</td><td colspan="2">Name: Dr David Williams</td></tr>
<tr><td>Signature</td><td>Date</td></tr>
</table>

13 DOCUMENT HISTORY

Version	Date	Edited by	Comments/Justification	Timing in relation to interim analysis/unblinding
0.1	08/04/2016	JB	First draft by Jennifer Bell	Prior to first interim analysis
0.2	06/05/2016	JB	Changes made to primary and secondary analysis. Minor amendments throughout.	Prior to first interim analysis
0.3	26/08/2016	JB	Transferred to new SAP template. Updated following changes to dummy tables and pending changes to protocol.	Prior to first interim analysis
0.4	07/09/2016	JB	Updated following review by Lucy Chappell.	Prior to first interim analysis
0.5	20/09/2016	JB	Updated following review by Pollyanna Hardy.	Prior to first interim analysis.
0.6	30/09/2016	JB	Updated following review by the CIG and further discussion with PH and LC.	Prior to first interim analysis.<br>JB was unblinded on 28/09/2016.
0.7	31/03/2017	JB	Reviewed by DMC.<br>Updated with comments on models for primary analysis, following feedback from John Norrie (DMC statistician)	After first interim analysis, prior to second interim analysis. No further unblinding since 28/09/2016.
0.8	30/10/2017	JB	Reviewed by Louise Linsell and Lucy Chappell.<br>Amended Adherence (section 4) and Repeated Measures analysis (section 7.2).<br>Moved to updated SAP template.	After second interim analysis, prior to third interim analysis. Unblinded on 10/04/ 2017.
0.9	01/11/2017	JB	Reviewed by Louise Linsell. Repeated Measures section edited.	After second interim analysis, prior to third interim analysis. No further unblinding since second interim analysis on 10/04/2017.
0.10	03/11/2017	JB	Reviewed by Lucy Chappell.	After second interim analysis, prior to third interim analysis. No further unblinding since second interim analysis on 10/04/2017.
0.11	09/02/2018	JB	Reviewed by TSC and CIG.<br>Adherence and Sensitivity Analysis sections amended.<br>Updated in line with protocol changes.<br>Removed section 2.10.1.<br>Other minor changes.	After second interim analysis, prior to third interim analysis. No further unblinding since second interim analysis on 10/04/2017.
0.12	14/03/2018	JB	Updated in line with protocol update (section 2.8).<br>Proof-read by Andrew King and reviewed by Louise Linsell. Added list of abbreviations, updated NPEU CTU logo. Minor grammatical and formatting changes.	After third interim analysis. No further unblinding since 24/01/18.

Version	Date	Edited by	Comments/Justification	Timing in relation to interim analysis/unblinding
1.0	15/03/2018	JB	Final version for sign-off.	After third interim analysis. No further unblinding since 24/01/18.
1.1	23/04/2018	JB	Updated following minor protocol amendment. Protocol version changed.	After third interim analysis. No further unblinding since 24/01/18.
1.2	10/08/2018	JB	Updated section 7.6 Significance levels following minor protocol amendment.	After third interim analysis. No further unblinding since 24/01/18.
