# Supplementary material for: Ursodeoxycholic acid versus placebo in the treatment of women with intrahepatic cholestasis of pregnancy (ICP) to improve perinatal outcomes: protocol for a randomised controlled trial (PITCHES)
Source: Trials. 2018 Nov 27;19:657. doi: 10.1186/s13063-018-3018-4 (PMC6260710; doi:10.1186/s13063-018-3018-4)
Supplement: Supplementary file 2 — Appendix 1. Participant information leaflet. (PDF 1717 kb) [file 13063_2018_3018_MOESM2_ESM.pdf]

PITCHES

Information Leaflet for Pregnant Women with ICP

Phase III trial in IntrahepaTic **CH**olestasis of pregnancy (ICP) to **E**valuate ur**S**odeoxycholic acid (UDCA) in improving perinatal outcomes.

We'd like to invite you to take part in our research study

- We appreciate this is a difficult time to think about taking part in research - we would like you to understand why the research is being done and what it would involve
- Please feel free to discuss the study with friends and family
- One of our team will go through the information with you and answer any questions you may have. We suggest this should take 10 minutes
- **Do ask if anything is unclear**
- **Joining the study is entirely up to you**
- **You will be given a copy of this information leaflet to keep**

Important things that you need to know

- **The standard of medical care you receive from your doctor and midwives will be the same whether you choose to take part in the study or not**
- We want to find the best way to treat a woman with ICP
- A drug called ursodeoxycholic acid (UDCA) is currently used to treat a woman with ICP, but we do not know if this is the best treatment – or if this helps babies
- The study visits fit into your normal clinical care
- You can stop taking part in the study at any time

Thank you for taking the time to read this information leaflet

MRC Medical Research Council

NHS National Institute for Health Research

The PITCHES study is funded by the Efficacy and Mechanism Evaluation (EME) Programme, an MRC and NIHR partnership

What is the purpose of the study?

Intrahepatic cholestasis of pregnancy (ICP) or obstetric cholestasis (OC) is a liver disorder which occurs during pregnancy. It affects 1 in 150 pregnancies each year in the UK, but the causes of ICP are not fully understood.

It can cause itching, mainly on the hands and feet, and may lead to marks on your skin from scratching even if there is no rash. This itching can be worse at night. ICP can be very uncomfortable, but the itchiness and abnormal liver function resolve themselves after the birth of your baby. The question that researchers want to answer is:

"If a woman has ICP, what are the consequences for the baby if she is treated with UDCA (or placebo)?"

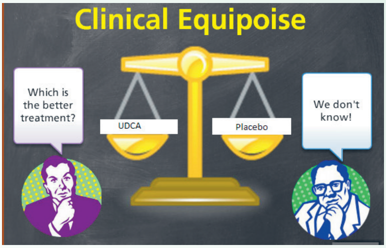

We understand that you will be worried how ICP might affect your baby. Research has shown that the condition may be linked to an increased risk of an early birth, both when it happens spontaneously (starting by itself) and by planned induction; and an increased likelihood of a baby passing meconium (baby poo) before birth so he/she may need to be looked after in a special care baby unit for a short time. There is evidence of a slight increase in risk of stillbirth in <2% of women with severe ICP.

In ICP the liver isn't working completely, this shows up in blood tests as an increase in bile acids. Bile acids are produced naturally by the body and are extremely important as they help you digest food, but if the body does not process them properly they can become damaging to the liver.

Researchers think that the large amount of bile acids passing through the placenta may have an effect possibly on the baby's heart, and may also damage the placenta, so it is thought that reducing bile acid levels may help protect the baby.

In our first small study (pilot study) we needed to see if women were happy to take part in this research and if UDCA helped them. We showed that it helped their itching a little (but not as much as they wanted) and it also seemed to help a woman's liver (shown by blood tests). The UDCA also helped to lower the bile acid levels for some women (but not all).

At the moment, not all women in the UK receive the same treatment for ICP: some doctors prescribe UDCA, and some do not. This is because doctors haven't got absolute proof to be sure of the best treatment to use. We need a large clinical study to see whether UDCA helps protect babies or not. At the end of the study, we hope to be able to give clear and definite guidance for doctors treating this condition.

Why have I been invited?

You have been diagnosed with ICP and are being cared for in one of the hospitals that is helping to answer the question.

We are asking 580 women with ICP, from 30 hospitals in the UK to get involved in the study which will run over 3 years.

Do I have to take part?

The study is voluntary which means you do not have to take part

Your decision must be the right choice for both you and your baby. Please chat with your family and friends, and ask any questions from your doctors/midwives. Also, if you decide to take part, but change your mind at any time, you can withdraw without giving a reason.

It is important that you understand what is happening at every stage. At each visit you will be given as much time as you need to discuss any concerns. You will also be given the name and contact number of someone you can talk to between visits.

If you decide **not** to take part, we will ask if you would let the research midwife at this hospital collect some information about your pregnancy and delivery from your notes. This will be used to help doctors see if there is any difference between your pregnancy and that of a woman taking part in the study.

What is the drug that is being tested?

The medicine that is being tested is ursodeoxycholic acid (UDCA), in tablets of 500mg.

It is a naturally occurring bile acid accounting for a small fraction of the total amount of bile acids in our bodies.

Unlike the other bile acids that are increased in the blood of women with ICP, UDCA is safe to the liver. In fact it is prescribed as a protective drug for the liver.

What will happen to me if I take part?

If you agree to take part, we will ask you to sign a consent form.

Your details will then be entered into a computer programme that will randomly select your treatment group which will either be UDCA or a placebo (which means that it looks like the medicine but is not active). There is an equal chance of you getting the active or non-active drug. The reason for doing this is because your doctor doesn't know whether to treat your ICP with UDCA or not and we hope that this research will give a clearer answer. By comparing the drug with the placebo in this 'blinded' study (also called a randomised controlled trial), neither you nor your doctor will know which group you are in, meaning that the results we get from the study will be as reliable as possible.

You will be prescribed 500 mg of UDCA or placebo, twice a day (2 tablets daily). Your doctor will increase the amount you are given by 500 mg up to a maximum of 2 g (4 tablets daily), if they think it is necessary, for example if your bile acids continue to rise. Also, your doctor will decrease the amount you are given if your bile acids go down.

The difference between normal clinical practice and taking part in this study:

Normal practice is to book a woman with ICP into a consultant-led team-based care. The first visit lasts 20-60 minutes and includes blood tests to monitor bile acids and liver enzymes, and often a tracing of the baby's heart (called a CTG or cardiotocography). Your doctor would decide whether to prescribe UDCA or not. A woman would then be seen weekly or fortnightly, each visit lasting about 20 minutes.

If you take part in this study, you or your doctor will not know whether you are taking UDCA or placebo.

In addition to the tests outlined above: we will ask you how bad your itch has been in the day before the visit.

At each visit we will ask you how you are getting on with taking your tablets. We will give you some extra tablets ('just in case') so we expect you to have tablets left in your bottle which we will count. This is important for our study records and helps us decide whether the tablets can help women with ICP or not.

You will not need extra blood tests outside normal clinical practice.

What are the possible risks and benefits of taking part?

There may be both risks and benefits in taking part which is why we feel it is important to do this study, to improve care for women with ICP.

UDCA is licensed for use, but not in pregnancy. However the manufacturer agrees that doctors may use it in pregnancy if they think it may be beneficial. Many doctors believe that it is safe to use and do prescribe it routinely in clinical practice for ICP. Our pilot study, and other similar studies, has suggested that UDCA may protect the unborn baby from poor outcomes, but the studies have not been large enough to be certain.

The study may not help you directly during this pregnancy, but the results will help us know if UDCA should be prescribed to women with ICP in the future.

If you would like to contact an organisation to discuss the inclusion of women with ICP in research studies generally we suggest that you contact ICP Support on 0781 7441726 or email *enquiries@icpsupport.org*.

What are the alternatives for treatment?

The alternative treatment for itching is applying lotions and creams. These may provide safe temporary relief of itching. A medicine called Chlorpheniramine is often prescribed for itching but there is no evidence that it can help reduce the itch nor reduce bile acid levels. Most doctors offer it to a woman because it has the side effect of making you feel sleepy and so may help at night when the itching is often more noticeable. Chlorpheniramine is also used to relieve hay fever symptoms (including in pregnancy) including red, itchy, watery eyes, sneezing and runny nose. Your doctor will discuss this with you. Aqueous and menthol cream that you put on your skin may help to soothe your skin but cannot reduce the bile acid levels.

What are the side effects of any treatment received when taking part?

If you experience loose stools (poo), please tell your doctor. If this is severe, stop taking your tablets and tell your doctor. However, your doctor will not know whether you are taking the UDCA or the placebo. We know that some pregnant women get loose stools anyway. Your doctor will decide with you whether to continue or stop the study treatment.

Can I take part in more than one study at a time?

Yes you can, as long as the doctors in charge of the studies agree, and this will depend on the medication involved and the research question being asked.

What if relevant new information becomes available?

We will inform you if any important new information becomes available during the course of the study that might mean the study is stopped. For example this might happen, if the answer became clear before the end of this study or if our question is answered by another study. If you change your mind about taking part and decide to withdraw, or your doctor considers you should withdraw, or the study is stopped, your care will continue following your hospital's preferred normal practice.

What will happen if I change my mind?

You are free to withdraw from the study at any time without giving a reason. We will ask if the information already collected about you can still be used. If you would still be happy to complete the itch charts at your normal clinical visits, we would use that information to help give us a clearer picture of ICP treatment within this study. Your ICP will still need treatment, so we will ask you to discuss your options with your doctor.

Will my taking part in this study be kept confidential?

All the information (data) collected about you during the course of the research will be kept strictly confidential and stored in a secure place. Some parts of your medical records will be looked at by the researchers, authorised persons from the co-sponsors, and representatives of regulatory authorities to make sure we carry out the study properly. Your data will be processed electronically to determine the outcome of this study. We may share anonymous data with others who are involved in similar research to support future decision-making.

What will happen to any samples I give?

The blood samples are part of routine clinical care, not the study so you would need to have the same blood tests for measuring your bile acid levels and liver enzymes whether you are take part in the study or not. The blood samples collected will be disposed of according to local laboratory policy.

Informing your family doctor

Your GP will be told that you are taking part in this study, but they will not know if you are taking UDCA or placebo. They will also be given a copy of this information leaflet.

What if there is a problem?

If at any stage you have any concerns about this study you should talk to the doctor or midwife who is leading the study in your hospital. You can also talk to the Patient Advice and Liaison Service (PALS) in your hospital (their contact details are on the back page of this leaflet). Information is also available on the study website [www.npeu.ox.ac.uk/PITCHES].

If you remain unhappy and wish to complain formally, you may use the normal NHS complaints procedure. Details of this procedure can be obtained from the following website: *http://www.nhs.uk/choiceintheNHS/Rightsandpledges/complaints/Pages/NHScomplaints.aspx*

What if something goes wrong?

If in the unlikely event you or your baby have been harmed by taking part in this study, you may have grounds for legal action and could seek compensation through the research co-sponsors, King's College London and Guy's and St Thomas' NHS Foundation Trust (GSTFT), who have appropriate insurance-related arrangements in place. If the harm is due to routine clinical treatment or negligence then the NHS indemnity arrangements will apply.

What will happen to the results of the research study?

The results of the study and a full report will be published in a medical journal and you will be sent a summary of the final results of the study. A copy of the full journal article can be requested from Women's Health at King's College London. Our results will also be put on the website ICP Support *www.icspsupport.org* . You will not be identified in any report or publication about the study.

Who is organising and funding this study?

The study is the responsibility of King's College London and Guy's and St Thomas' NHS Foundation Trust (GSTFT) and is being run by the Clinical Trial Unit (CTU) at the National Perinatal Epidemiology Unit (NPEU), which is a department of Oxford University. It is funded by the National Institute of Health Research (NIHR) Efficacy Mechanisms & Evaluation (EME) programme (ref. 12/164/16). None of the researchers receive payments for taking part in the study; and your doctor will not be paid for enrolling you onto the study.

Who has reviewed the research?

All research in the NHS is looked at by an independent group of people, called a Research Ethics Committee. This study has been approved by the Essex Committee of East of England which means that they are satisfied that your rights will be respected; that any risks have been reduced to a minimum and balanced against possible benefits; that you have been given sufficient information on which to make an informed decision to take part or not; and that they believe the study is fair.

The ethics committee reviews a protocol before the study is allowed to start. Their job is to ensure that the risks of being in the study are not greater than the potential benefit.

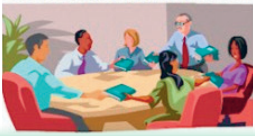

How have patients and the public been involved in this study?

Members of ICP Support have helped in the design of this study and gave us feedback about this information leaflet.

www.icpsupport.org

Local contacts

Principal Investigator

{_LEAD_}

Local Research Midwife

{_MIDWIVES_}

Patient Advice and liaison services (PALS)

{_PALS_}

PITCHES Study Coordinator

Anne Smith
NPEU Clinical Trials Unit
National Perinatal Epidemiology Unit
Nuffield Department of Population Health, University of Oxford
Old Road Campus, Oxford OX3 7LF

01865 289735 pitches@npeu.ox.ac.uk www.npeu.ox.ac.uk/pitches

EudraCT Number: 2014-004478-41 REC Reference: 15/EE/0010 ISRCTN 91918806

King's College London · Guy's and St Thomas' NHS Foundation Trust · npeu Clinical Trials Unit · University of Oxford
