# Supplementary material for: Ursodeoxycholic acid versus placebo in the treatment of women with intrahepatic cholestasis of pregnancy (ICP) to improve perinatal outcomes: protocol for a randomised controlled trial (PITCHES)
Source: Trials. 2018 Nov 27;19:657. doi: 10.1186/s13063-018-3018-4 (PMC6260710; doi:10.1186/s13063-018-3018-4)
Supplement: Supplementary file 3 — Appendix 2. Consent form. (PDF 366 kb) [file 13063_2018_3018_MOESM3_ESM.pdf]

PITCHES

Consent Form

Please complete in black ballpoint pen

Phase III trial in IntrahepaTic CHolestasis of pregnancy (ICP)
to Evaluate urSodeoxycholic acid (UDCA) in improving perinatal outcomes

Hospital Name: ______________________________

Chief Investigator: Dr Lucy Chappell

Study Number: ☐☐☐☐☐

		**Please initial box**
1.	I confirm that I have read and understand the Participant Information Leaflet (Version 2 dated 12Feb15) for this study and have had the opportunity to ask questions which have been answered satisfactorily.	☐
2.	I understand that participation in this study is voluntary and that I am free to withdraw from the study at any time without giving any reason, and without my present or future medical care or legal rights being affected.	☐
3.	I understand that relevant sections of medical records and data collected during the study may be looked at by the study coordinating centre in Oxford, the co-sponsors, or this NHS Trust. I give permission for these individuals to have access to these records where it is relevant to taking part in this research.	☐
4.	I understand that information held and managed by the Health and Social Care Information Centre and other central UK NHS bodies will be used in order to help contact me or provide information about my health status, and that of my baby. This is on the understanding that all information will be treated confidentially.	☐
5.	I agree that personal identifiable information will be collected, stored and used to follow my progress through the study and enable follow-up. This is on the understanding that all information will be treated confidentially.	☐
6.	I agree to my GP being informed of my participation in the study.	☐
7.	I agree to take part in the study.	☐

Name of participant	Name of health professional taking consent
Signature	Signature
DD / MM / YY	DD / MM / YY

PITCHES Trial
NPEU Clinical Trials Unit, National Perinatal Epidemiology Unit
Nuffield Department of Population Health, University of Oxford
Old Road Campus, Oxford, OX3 7LF
T: 01865 289735 F: 01865 289740
E: pitches@npeu.ox.ac.uk W: www.npeu.ox.ac.uk/pitches

EudraCT Number 2014-004478-41, ISRCTN 91918806, REC Reference 15/EE/0010
Version 2, 12 February 2015

Guy's and St Thomas' NHS
NHS Foundation Trust

KING'S College LONDON

npeu
Clinical Trials Unit

UNIVERSITY OF OXFORD

The PITCHES study is funded by the Efficacy and Mechanism Evaluation (EME) Programme, a Medical Research Council (MRC) and National Institute for Health Research (NIHR) partnership

WHITE copy to PITCHES Coordinating Centre; GREEN copy to participant; YELLOW copy to PITCHES Data Collection File; PINK copy to participant's notes
